# Deubiquitinase DUB3 Regulates Cell Cycle Progression via Stabilizing Cyclin A for Proliferation of Non-Small Cell Lung Cancer Cells

**DOI:** 10.3390/cells8040297

**Published:** 2019-03-31

**Authors:** Bin Hu, Tanggang Deng, Hongchang Ma, Yan Liu, Peifu Feng, Dong Wei, Neng Ling, Ling Li, Siyuan Qiu, Lin Zhang, Bo Peng, Jing Liu, Mao Ye

**Affiliations:** 1 Molecular Science and Biomedicine Laboratory, State Key Laboratory for Chemo/Biosensing and Chemometrics, College of Biology, College of Chemistry and Chemical Engineering, Collaborative Innovation Center for Molecular Engineering for Theranostics, Hunan University, Changsha 410082, China; 18390940720@163.com (B.H.); dengtanggang@163.com (T.D.); 15116234699@163.com (H.M.); ellie_lsy@163.com (Y.L.); peifufeng@hnu.edu.cn (P.F.); weidonglyn@126.com (D.W.); 15773467660@163.com (N.L.); 15974250722@163.com (L.L.); 15575833145@163.com (S.Q.); zhlin@hnu.edu.cn (L.Z.); pengbo2017@hnu.edu.cn (B.P.); 2 School of Life Sciences, Central South University, Changsha 410078, China

**Keywords:** deubiquitination, cell cycle, DUB3, cyclin A

## Abstract

The deubiquitinase DUB3 is frequently overexpressed in non-small cell lung cancer (NSCLC) and contributes to its malignant phenotype. However, the underlying molecular mechanism of DUB3 in NSCLC is largely unknown. In this study, we report that DUB3 regulates cell cycle progression by deubiquitinating cyclin A that links to proliferation of NSCLC cells. We found that knockdown of DUB3 decreases cyclin A levels, whereas overexpression of DUB3 strongly increases cyclin A levels. Mechanistically, DUB3 interacts with cyclin A, which removes the polyubiquitin chains conjugated onto cyclin A and stabilizes the cyclin A protein. Furthermore, we demonstrate that DUB3 regulates cell cycle progression by stabilizing cyclin A, because ablation of DUB3 arrests cell cycle from G0/G1 to S phase and the resulting effect can be rescued by introducing cyclin A into NSCLC cells. Functionally, we found that the effect of DUB3 on cyclin A mediates proliferation of NSCLC cells. Moreover, a significant correlation between DUB3 abundance and cyclin A expression levels were also found in NSCLC samples. Taken together, these results reveal that DUB3 functions as a novel cyclin A regulator through maintaining cyclin A stability, and that the DUB3-cyclin A signaling axis plays a critical role in cell cycle progression for proliferation of NSCLC.

## 1. Introduction

Lung cancer is a frequently diagnosed malignancy with the highest morbidity and mortality around the world. According to the latest Global Cancer Statistics 2018, lung cancer accounts for 11.6% of the total diagnosed cancer cases, and for 18.4% of the total cancer-related deaths [1]. Non-Small Cell Lung Cancer (NSCLC) is the most common type of lung cancer, making up about 80–85% of all cases. Despite tremendous efforts in developing new therapeutic strategies for NSCLC, the overall 5-year survival rates less than 15% [2]. Therefore, it is urgent to elucidate the molecular mechanism of tumorigenesis for effective manipulation of NSCLC.

It is well documented that ubiquitin-mediated protein degradation plays an essential role in a multitude of cancer-related cellular process. Ubiquitination is a reversible process and ubiquitin moieties conjugated onto protein can be removed by deubiquitinases (DUBs). The dynamic balance between ubiquitination and deubiquitination regulates the stability of most cellular protein. More than 100 DUBs are encoded in the human genome, which are classified into six different subfamilies, namely ubiquitin carboxy-terminal hydrolases (UCHs), ubiquitin-specific proteases (USPs), Jab1/Pad1/MPN domain-associated metallopeptidases (JAMMs), ovarian-tumor proteases (OTUs), Machado-Joseph disease protein domain proteases and monocyte chemotactic protein-induced protein (MCPIP) [3]. Growing evidence shows that DUBs are involved in various cellular functions, including cell cycle progression. Aberrant expression/mutations of DUBs are frequently found in human cancer.

DUB3 (also termed USP17) belongs to the USP subfamily, and is an immediate early gene whose transcription can be rapidly induced by cytokines [4]. Previous studies show that DUB3 expression is cell cycle regulated [5], and is required for cell cycle progression [6]. DUB3 overexpression has been observed in NSCLC [7], ovarian cancer [8], prostate cancer [9], osteosarcoma [10] and glioma [11]. In particular, DUB3 expression levels correlates with NSCLC recurrence and metastasis [12].

Cyclin A, a member of the cyclin family, plays a crucial role during cell cycle progression [13]. Specifically, cyclin A promotes G1/S transition through interacting with and activating CDK2. Moreover, it is also involved in the G2/M transition through binding to and activating CDK1 [14]. Growing evidence suggests that the cellular cyclin A abundance is tightly regulated at transcriptional and post-transcriptional levels. E3 ligase APC/C complex has been found to target cyclin A for ubiquitin-mediated degradation [15]. By contrast, deubiquitinase USP37 can be activated by CDK2, which in turn stabilizes cyclin A through removing degradative polyubiquitin chains from cyclin A [16].

In this study, we identify DUB3 as a new regulator of cyclin A. DUB3 binds to cyclin A and promotes its deubiquitination and stabilization. We also demonstrate that DUB3-cyclin A axis is critical for G1/S transition during cell cycle progression. The ablation of DUB3 arrests cell cycle from G1 to S phase. As a result, DUB3 promotes proliferation of NSCLC cells. Meanwhile, we also found that DUB3 and cyclin A levels are positive correlated in clinical samples of NSCLC patients. Taken together, our findings reveal a novel role of DUB3 in the proliferation of NSCLC, implicating DUB3 as a promising target for NSCLC diagnosis and treatment.

## 2. Materials and Methods

### 2.1. Cell Culture and Reagents

A549 and H1299 cells were cultured in 1640 media (GIBCO) supplemented with 10% FBS at 37 °C in 5% CO_2_ humidified incubators. MG132 (cat. C2211) was purchased from Sigma-Aldrich (St. Louis, MO, USA). Thymidine (cat. 6060) was acquired from Millipore (Burlington, MA, USA). GeneMut siRNA transfection reagent (cat. SL100568) was purchased from SignaGen Laboratories (Rockville, MD, USA). LipoMax plasmid transfection reagent (cat. 17052012) was purchased from SUDGEN (Bellevue, WA, USA). M-PER lysis buffer (cat. 78501), Dynabeads (cat. 10004D) and Clean-blot™ IP detection reagent (HRP) (cat. 21230) were purchased from Thermo Fisher (Waltham, MA, USA). RIPA buffer (cat. P0013B) and Cell cycle and apoptosis analysis kit (cat. C1052) were purchased from Beyotime (Shanghai, China). The protease inhibitor cocktail (cat. B14012) and Cell Counting Kit-8 solution (CCK-8, cat. B34304) were purchased from Bimake (Houston, TX, USA). HRP-labeled goat anti-mouse (cat. GB23301) and goat anti-rabbit secondary antibody (cat. purGB23303) were purchased from Servicebio (Wuhan, China).

### 2.2. Plasmids

Full-length DUB3 was PCR-amplified from human cDNA and subcloned into pCMV-Tag2B (with the Flag tag) to create Flag-tagged DUB3 expression plasmid. The catalytically inactive DUB3 mutant (C89S) was generated using PCR. pCMV-Tag2B-cyclin A plasmid was purchased from Vigene Biosciences (Rockville, MD, USA). All constructs were sequenced before use.

### 2.3. Antibodies

Anti-DUB3 (cat. #PA5-44961) antibody was purchased from Invitrogen (Waltham, MA, USA). Anti-cyclin A (cat.ab38) antibody was from Abcam (Cambridge, England). Anti-Ub (cat. 550944) and anti-cyclin A (cat.554174) antibodies were from BD Biosciences (Franklin Lakes, NJ, USA). Anti-Flag (cat. M185-3L) antibody was from MBL (Tokyo, Japan). Anti-GAPDH (cat.AT0002) antibody was from Cmctag (Milwaukee, WI, USA).

### 2.4. Protein Half-Life Assays

Cells were treated with cycloheximide (50 µg/mL) for various periods to block protein synthesis. Crude extracts were prepared and protein levels were assessed by Western blot analysis.

### 2.5. Synchronization of Cells

To synchronize A549 cells at the G1/S border, cells were treated with 2 mM thymidine for 17 h. Cells were released from the block by adding complete growth medium. After 8 h, thymidine was added to the medium to a final concentration of 2 mM, and cells were cultured for an additional 17 h. Cells were then rinsed twice with PBS and cultured in complete growth medium. After cells were released by adding complete culture media for 2 h, cells were collected and analyzed by flow cytometry and western blotting.

### 2.6. Cell Cycle Analysis

Cells were seeded in 6-well plates at 3 × 10^5^ cells per well for 72 h prior to analysis. The cells were then harvested and washed in (Dulbecco’s Phosphate-Buffered Saline) DPBS, followed by fixation with pre-cooled 70% ethanol and stored at −20 °C overnight. Next, the cells were treated with RNase A, stained with propidium iodide according to the manufacturer’s protocol. The stained cells were detected using flow cytometry (BD FACSVerse^TM^, Franklin Lakes, NJ, USA).

### 2.7. Real-Time PCR

Total RNA was extracted with Eastep^®^ (Promega, Madison, WI, USA). RNA (1 μg) was reverse-transcribed in a 20 μL reaction using the FastQuant RT Super Mix Kit (Tiangen, Beijing, China). After reverse transcription at 42 °C for 15 min and inactivation by incubating samples at 95 °C for 3 min, the RT reaction was diluted. cDNA was used for RT-PCR or real-time PCR assay. Primer sequences were as follows:

2—DUB3-F, GAGCACTTGGTGGAAAGAGC

3—DUB3-R, TGATGGTTCTTCTTCATCCCACA

4—GAPDH-F, CAGCGACACCCACTCCTCCACCTT

5—GAPDH-R, CATGAGGTCCACCACCCTGTTGCT

6—CyclinA-F, GCCATTAGTTTACCTGGACCCAGA

7—CyclinA-R, CACTGACATGGAAGACAGGAACCT

### 2.8. RNA Interference and Lentivirus Transduction

The sequence of the DUB3 siRNA was 5′-GAAAUUCCUUCAAGAGCAA-3′). The sequence of control siRNA was 5′-TTCTCCGAACGTGTCACGTTTC-3. These siRNAs were synthesized by Shanghai GenePharma (Shanghai, China). Transfection was carried out according to the manufacturer’s protocol. After 48 h, cells were washed with PBS, lysed directly with M-PER lysis buffer and protein levels were assessed by Western blot analysis. To stably knock down endogenous DUB3 expression in some case, we used lentivirus packing shRNA expression vector (purchased from Shanghai GenePharma) to infect cells. Target cells were infected with lentivirus for 24–48 h according to manufacturer’s instruction. The DUB3 shRNA target sequence was 5′-GCAGGAAGATGCCCATGAATT-3′. The control shRNA sequence was 5′-TTCTCCGAACGTGTCACGT-3′.

### 2.9. Ubiquitination Assay

A549 cells were infected with the indicated lentiviruses for 72 h, or transfected with specified plasmids for 24 h followed by treatment with 20 μM MG132 for 6 h, washes with PBS, and lysis in RIPA buffer containing a protease inhibitor cocktail. The lysates were transferred into a 1.5 mL tube and placed on a hot plate immediately to boil for 10 min. Then, the lysates were centrifuged to obtain protein, which was incubated with anti-cyclin A antibody overnight, followed by treatment with protein A/G beads for an additional 1 h at room temperature. Then, the beads were washed three times with PBS buffer containing 1‰ Tween-20 (PBST). After the proteins were released from the beads by boiling the beads in SDS/PAGE sample buffer, they were analyzed using immunoblotting with anti-Ub antibody.

### 2.10. Cell Proliferation Assay

A549 cells infected with indicated lentiviral shRNA constructs for 48 h were seeded into a 6-well plate at an optimized density of 3 × 10^5^ cells per well. After cyclin A or empty vector were transfected into indicated cells for another 24 h, cells were seeded into a 96-well plate at an optimized density of 5 × 10^3^ cells per well in quadruplicate. At the indicated time-points, the complete medium containing 10% CCK-8 was added to each well and incubated at 37 °C for another 1 h. The absorbance of the individual wells was determined at 450 nm. Each experiment was repeated at least three times.

### 2.11. Colony Formation Assay

A549 cells were infected lentiviral shRNAs and transfected with control pCMV-Tag2B plasmid or pCMV-Tag2B-cyclin A construct for 24 h, and then plated in 6-well plates at 5000 cells/well or 7000 cells/well, respectively. Cells were cultured in the selection medium (800 μg/mL G418, Sigma-Aldrich). After a 2-week selection, cells were fixed with 4% paraformaldehyde for 30 min and stained with 0.1% crystal violet, and the number of colonies were counted.

### 2.12. Immunohistochemistry

The tissue microarrays containing forty-five paired NSCLC samples and their corresponding adjacent normal tissues (cat. LC10012b) were purchased from US Biomax (Derwood, MD, USA). The arrays were treated with antigen retrieval buffer (0.01 M sodium citrate, pH 6.0). Specifically, DUB3 or cyclin A antibody was applied overnight at room temperature at a dilution of 1:100. Slides were then incubated with secondary antibody for 1 h at room temperature. Next, slides were incubated with DAB until desired stain intensity was observed, followed by counterstain in nucleus with Hematoxylin staining solution for 3 min, treated with the differentiate solution for a few seconds and returned blue with ammonia. Finally, slides were dehydrated successively in gradient ethanol, cleared in xylene for 5 min and mounted with resin mounting medium. After staining, slides were scanned by Pannoramic MIDI digital slide scanner (3DHISTECH).

Levels of DUB3 and cyclin A expression in lung cancer tissue specimens from NSCLC patients were reviewed and scored under a light microscope by two independent pathologists who were not aware of the clinicopathological data. If there was a discrepancy, a consensus interpretation was reached under a two-headed microscope. The DUB3 and cyclin A expression was quantified by a visual grading system (0–3) based on the intensity of cytoplasm and nuclear staining as follows: Grade 0, no immunoreactivity; grade 1, weak immunoreactivity slightly stronger than background staining; grade 2, clear immunoreactivity in more than half of the cancer cells; and grade 3, strong immunoreactivity as dark as the nuclear counterstain in the majority of cancer cells. Finally, the cases were classified into two different groups: low expression (grade 0 and 1) and high expression (grade 2 and 3). The X^2^ test was used for statistical analysis of the correlation between DUB3 and cyclin A.

## 3. Results

### 3.1. DUB3 Regulates the Protein Levels of Cyclin A

**Figure 1:**
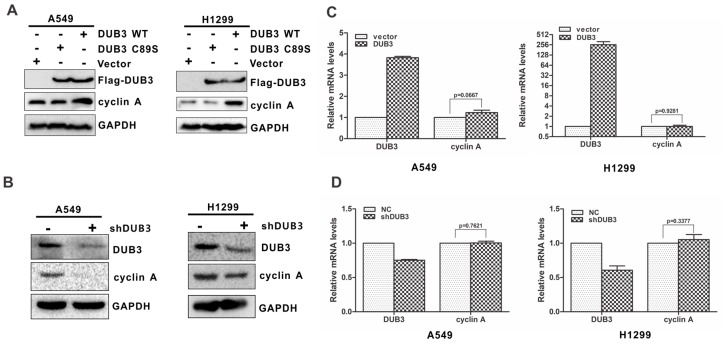
DUB3 influences the protein level of cyclin A. (**A**) A549 and H1299 cells were transfected with the indicated constructs. Total protein was extracted and subjected to Western blotting using anti-Flag, anti-cyclin A or anti-GAPDH antibody. (**B**) A549 and H1299 cells were infected with indicated lentiviral shRNA. The resulting cell extracts were analyzed using Western blotting with anti-DUB3, anti-cyclin A or anti-GAPDH antibody. (**C**) A549 and H1299 cells were transfected with a plasmid encoding Flag-DUB3 or an empty vector control. Total RNA was isolated and subjected to real-time PCR. (**D**) A549 and H1299 cells were infected with indicated lentivirus encoding the DUB3 shRNA or control shRNA. Total RNA was isolated and subjected to real-time PCR. The error bars represent the SD of triplicate measurements.

Growing evidence shows a strong link between cyclin A overexpression and NSCLC [17,18]. To clarify the potential mechanism by which DUB3 promotes the proliferation and tumorigenesis of NSCLC, we examined whether DUB3 would affect the levels of cyclin A. DUB3 was introduced into A549 and H1299 cells. Intriguingly, DUB3 overexpression significantly increased the endogenous cyclin A levels compared with the vector control (Figure 1A). By contrast, overexpression of a catalytically inactive DUB3 mutant (C89S) had no effect on cyclin A levels (Figure 1A), indicating that DUB3-mediated upregulation of cyclin A depends on the function of DUB3 as a deubiquitinating enzyme. To further valid the effect of DUB3 on cyclin A, we performed a loss-of-function analysis using DUB3-specific short hairpin RNAs (shRNAs) in A549 and H1299 cells. As predicted, DUB3 knockdown dramatically decreased the cyclin A levels (Figure 1B). Similar results were obtained using DUB3-specific interfering RNAs (siRNAs) in A549 and H1299 cells (Figure S1A). 

To determine whether the effect of DUB3 on cyclin A is regulated at the transcriptional levels, we employed real-time PCR to assess the expression of cyclin A mRNA. We found that neither the overexpression of nor the knockdown of DUB3 altered cyclin A mRNA levels (Figure 1C,D and Figure S1B). These findings suggest that DUB3 positively regulates cyclin A at the protein level, but not at the transcriptional level.

### 3.2. DUB3 Regulates Cyclin A Levels in A Proteasome-Dependent Manner

**Figure 2:**
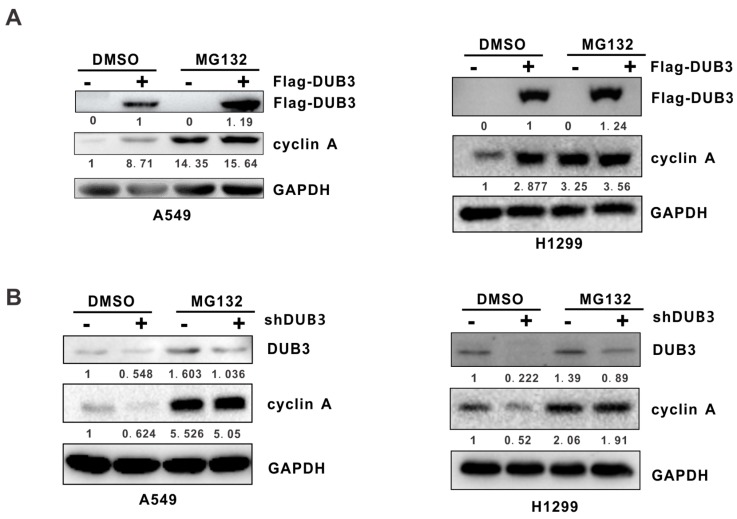
DUB3 regulates cyclin A levels via the proteasome pathway. (**A**) A549 and H1299 cells transfected with Flag-DUB3 plasmid or the control plasmid were treated with DMSO or MG132 (20 μM) for 6 h. Expression of the indicated proteins were examined by Western blotting using the indicated antibodies. (**B**) A549 and H1299 cells infected with the indicated lentiviral shRNAs were treated with DMSO or MG132 (20 μM) for 6 h, and the indicated proteins were analyzed by Western blotting.

Most intracellular proteins are degraded by the proteasome system. As DUB3 affected cyclin A at the protein level, we speculated that DUB3 mediated the regulation of cyclin A through the proteasome pathway. To test this hypothesis, A549 and H1299 cells transfected with an empty vector or a plasmid expressing Flag-DUB3 were treated with the proteasome inhibitor MG132, and the endogenous cyclin A was examined by Western blotting. As expected, in the absence of MG132, overexpression of DUB3 led to up-regulation of cyclin A (Figure 2A). However, MG132 pretreatment effectively abolished DUB3-mediated regulation of cyclin A (Figure 2A). Similar results were also obtained following knockdown of DUB3 using DUB3-specific shRNA (Figure 2B) or siRNA (Figure S2) in A549 and H1299 cells. Taken together, these results suggest that DUB3 maintains the steady-state levels of cyclin A by blocking its proteasomal degradation.

### 3.3. DUB3 Interacts with Cyclin A

**Figure 3:**
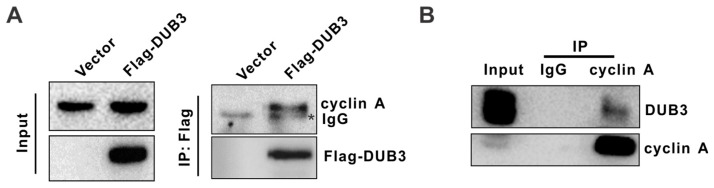
DUB3 interacts with cyclin A. (**A**) A549 cells were transfected with Flag-DUB3 plasmid or the control plasmid. Total cell lysates were subjected to immunoprecipitation with anti-Flag antibodies. The immunoprecipitates were then probed with anti-Flag or anti-cyclin A antibodies. (* IgG) (**B**) A549 cell lysates were subjected to immunoprecipitation with control IgG or anti-cyclin A antibodies. The immunoprecipitates were then probed with anti-DUB3 or anti-cyclin A antibodies.

Given that DUB3 regulates cyclin A levels, we asked if DUB3 physically interacts with cyclin A. To this end, Flag-DUB3 plasmids were transfected into A549 cells, and coimmunoprecipitation (co-IP) was performed using anti-Flag antibodies. We found that cyclin A was co-immunoprecipitated by anti-Flag antibodies in DUB3-overexpressing cells but not in negative control cells transfected with the same amount of empty vector (Figure 3A). Meanwhile, we examined whether endogenous DUB3 interacts with cyclin A in A549 cells. As shown in Figure 3B, DUB3 was detected in the anti-cyclin A immunoprecipitates but not in the isotype-matched negative control IgG complexes. Collectively, these results suggest that DUB3 interacts with cyclin A.

### 3.4. DUB3 Stabilizes Cyclin A by Deubiquitination

**Figure 4:**
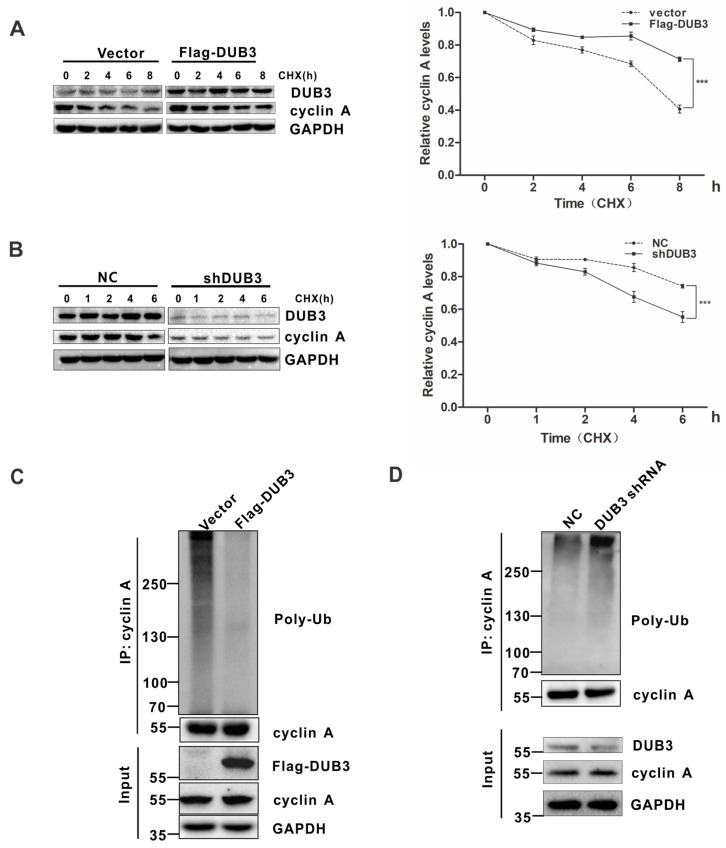
DUB3 stabilizes cyclin A through deubiquitination. (**A**) A549 cells transfected with the indicated plasmids were treated with 50 μg·mL^−1^ cycloheximide (CHX), collected at the indicated time points, and immunoblotted with anti-DUB3, anti-cyclin A, and anti-GAPDH antibodies. Quantification of the cyclin A levels relative to GAPDH expression is shown. Data represent the mean (± S.D.) of three independent experiments (*** *p* < 0.001). (**B**) A549 cells infected with the indicated lentiviral shRNAs were treated with 50 μg·mL^−1^ CHX and then collected at the indicated time points for Western blot analysis. Quantification of the cyclin A levels relative to GAPDH expression is shown. Data represent the mean (± S.D.) of three independent experiments (*** *p* < 0.001). (**C**,**D**) A549 cells either transfected with the indicated constructs (**C**) or infected with the indicated lentiviral shRNAs (**D**) were treated with MG132 (20 μM) for 6 h before harvest. Cyclin A was immunoprecipitated with anti-cyclin A antibodies, and the immunoprecipitates were probed with anti-Ub or anti-cyclin A antibodies.

Since DUB3 prevents cyclin A from proteasome-mediated degradation, we hypothesized that DUB3 might affected cyclin A protein stability. To address this, A549 cells transfected with or without DUB3 were treated with cycloheximide (CHX) to inhibit protein biosynthesis, and protein extracts obtained at indicated time points were analyzed. As shown in Figure 4A, overexpression of DUB3 significantly prolonged the half-life of endogenous cyclin A. By contrast, in cells depleted of DUB3, the half-life of cyclin A was significantly decreased (Figure 4B and Figure S3A), indicating that DUB3 regulates the stability of cyclin A protein.

To further understand the underlying mechanism that DUB3 stabilizes cyclin A, we measured the levels of cyclin A polyubiquitination in A549 cells. We found that ectopic expression of DUB3 significantly reduced the polyubiquitination of cyclin A (Figure 4C). Conversely, knockdown of endogenous DUB3 using shRNAs or siRNAs caused a significant increase in cyclin A polyubiquitination (Figure 4D and Figure S3B). Collectively, these results suggest that DUB3 stabilizes cyclin A through deubiquitination.

### 3.5. DUB3 Regulates G1/S Transition in A Cyclin A-Dependent Manner

**Figure 5:**
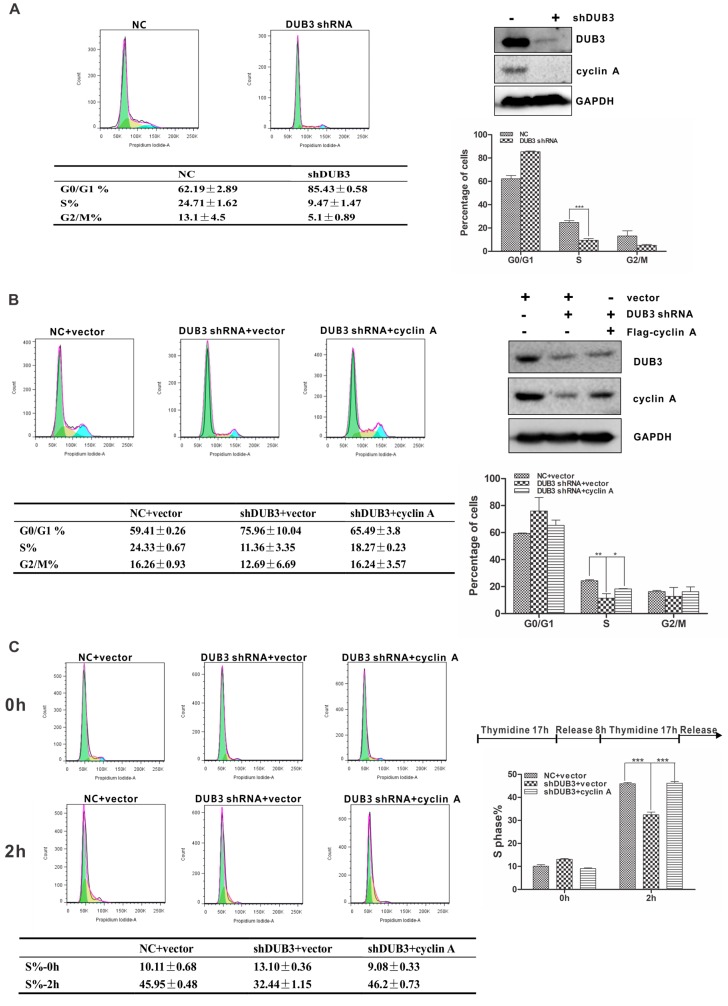
DUB3 regulates the G1/S transition in a cyclin A-dependent manner. (**A**) A549 cells infected with the indicated lentiviral shRNAs were stained with propidium iodide and analyzed using flow cytometry. Data represent the mean (± S.D.) of three independent experiments (*** *p* < 0.001). (**B**) A549 cells infected with the indicated lentiviral shRNAs with or without ectopic expression of cyclin A were stained with propidium iodide and analyzed using flow cytometry. Data represent the mean (± S.D.) of three independent experiments (* *p* < 0.05 and ** *p* < 0.01). (**C**) A549 cells stably expressing indicated DUB3 shRNA were synchronized by a double-thymidine block. The released cells were then harvested at the indicated time points and analyzed by flow cytometry. The percentage of S-phase cells is shown. Data represent the mean (± S.D.) of three independent experiments (*** *p* < 0.001).

It is well known that cyclin A plays an essential role in the G1/S transition of cell cycle. To test if DUB3 affects cell cycle progression, we knocked down DUB3 and examined cell cycle distribution of A549 cells by flow cytometric analysis following with Propidium Iodide (PI) staining. Compared with the control cells, the percentage of S-phase cells was significantly decreased in DUB3-silenced A549 cells (Figure 5A and Figure S4). Interestingly, the effect of DUB3 ablation on cell cycle can be rescued by instruction of ectopic cyclin A (Figure 5B). To further confirm this finding, A549 cells were synchronized at the G1/S border by double thymidine block and release. Likewise, DUB3 knockdown in A549 cells delayed entry into S phase, whereas the resulting effect could be restored by introducing cyclin A into DUB3-depleted cells (Figure 5C). Collectively, these results indicate that DUB3 regulates G1/S transition in a cyclin A-dependent manner.

### 3.6. DUB3 Promotes Proliferation of NSCLC Cells Through Cyclin A

**Figure 6:**
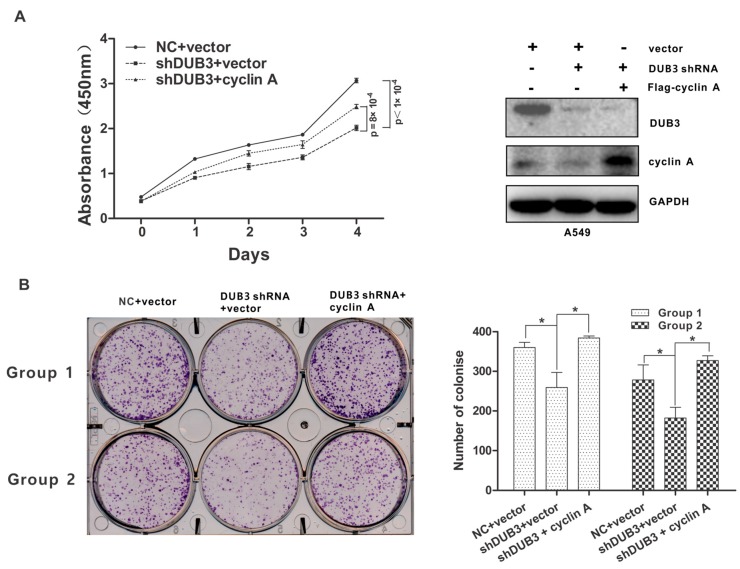
DUB3 promotes NSCLC cell proliferation via cyclin A. (**A**,**B**) A549 cells were infected with the indicated lentiviral shRNAs and then transfected with the indicated constructs. Cell proliferation was monitored using CCK-8 assays at the indicated time points (**A**). Colony formation assay was performed (**B**). Colonies were visualized by light microscopy and quantified. Cell seeding density was 7000 cells per well (Group 1) and 5000 cells per well (Group 2), respectively. Statistical significance was determined by a two-tailed, unpaired Student’s t test. Data represent the mean (± S.D.) of three independent experiments (* *p* < 0.05).

Previous studies have demonstrated that DUB3 was frequently overexpressed in NSCLC tissues and promotes proliferation of NSCLC cells [7,12]. To investigate if DUB3 affects cell proliferation via acting on cyclin A, we conducted a cell proliferation assay using CCK-8. Consistent with previous reports, DUB3 knockdown inhibited proliferation of A549 cells, whereas cyclin A restoration reversed the effect of DUB3 depletion (Figure 6A and Figure S5). Similar results were obtained by colony formation assay (Figure 6B), indicating that DUB3 mediates cell proliferation through cyclin A.

### 3.7. DUB3 Is Overexpressed and Positively Correlates with Cyclin A in Clinical Samples of NSCLC

**Figure 7:**
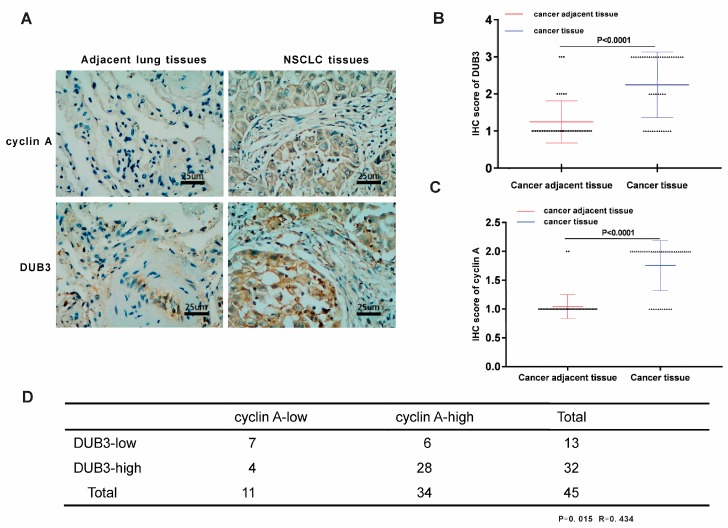
DUB3 and cyclin A are overexpressed and positively correlated in NSCLC samples. (**A**) Representative images from immunohistochemical staining of DUB3 and cyclin A in adjacent lung tissues and NSCLC tissues were shown. Scale bars, 25 μm. (**B**,**C**) DUB3 (**B**) cyclin A (**C**) protein expression status in adjacent lung tissues and NSCLC tissues. (**D**) A summary of immunohistochemical staining for the correlation between DUB3 and cyclin A in NSCLC. The statistical analysis was determined by a X^2^ test.

To determine the relevance of regulation of cyclin A by DUB3 in patients, we performed immunohistochemical staining of cyclin A and DUB3 on 50 pairs of NSCLC tissue microarrays. High expression of DUB3 and cyclin A was observed in 70% (35 of 50) of NSCLC, whereas only 18% (9 of 50) and 4% (2 of 50) of the corresponding adjacent lung tissues exhibited high expression of DUB3 and cyclin A (Figure 7A–C), respectively. This suggests that both DUB3 and cyclin A are overexpressed in NSCLC. Representative positive and negative stains of cyclin A and DUB3 in NSCLC tissues and adjacent lung tissues are shown in Figure 7A. Moreover, a significant positive correlation (R = 0.3333, *p* = 0.018) between DUB3 and cyclin A protein levels was observed in these NSCLC tissues (Figure 7D), in which 80% (28 of 35) of the tumor samples with high DUB3 expression also exhibited high cyclin A. Thus, these results in NSCLC tissues support our proposed mechanism based on cell culture experiments that expression of DUB3 contribute to maintain cyclin A levels. Overexpression of DUB3 may promote cyclin A stability and enhanced tumorigenesis in NSCLC patients.

## 4. Discussion

In this study, we identified DUB3 as a cyclin A deubiquitinase. Our data showed that overexpression of DUB3 increased cyclin A levels, whereas depletion of DUB3 decreased the cyclin A levels. Functionally, DUB3 drives cell cycle progression and mediates proliferation of NSCLC cells via stabilizing cyclin A. Thus, DUB3 is a novel regulator of cyclin A.

DUB3 functions as a deubiquitinase, and its activity can be regulated at the post-translational level. Previous studies have demonstrated that CDK4/6-mediated phosphorylation of DUB3 on Ser41 is essential for activation of the enzymatic function, which contributes to deubiquitinating and stabilizing SNAIL1, a key factor in epithelial-mesenchymal transition (EMT). The CDK4/6 inhibitor palbociclib (PD0332991) can reduce triple-negative breast cancer (TNBC) metastasis via inducing the inactivation of DUB3 [19]. As abnormal activation of CDK4/6 also frequently occurs in NSCLC [20], it would be interesting to investigate if the stability of cyclin A also depends on CDK4/6-mediated activation in DUB3.

Numerous studies show that DUBs are involved in cell cycle regulation. CYLD regulates entry into mitosis through targeting Plk1 [21]. USP3 functions as a chromatin modifier required for S phase progression [22]. USP11 stabilizes p21 and plays a critical role in G1/S transition [23]. Here, we showed that DUB3 mediated the regulation of cell cycle progression. Loss of DUB3 resulted in cell cycle arrest at the G1/S phase, which is consistent with previous reports that DUB3 is required for G1-S progression [6,24]. Furthermore, we found that cyclin A restoration reversed the effect of DUB3 depletion on the cell cycle, indicating that DUB3 depends on cyclin A to regulate the G1/S transition. Of note, DUB3 is also shown to stabilize the dual specificity (Tyr/Thr) phosphatase Cdc25A for G1/S transition [24]. It is well known that the Cdc25A phosphatase is a crucial activator of the CDK-cyclin complex for cell cycle progression [25]. At the end of G1, Cdc25A drives entry into S phase through activating the CDK2-cyclin A/E complex [26]. We speculate that DUB3 may perform dual effects on Cdc25A and cyclin A, and accelerates G1/S transition. Further studies are needed to fully understand the synergistic mechanism of DUB3 on Cdc25A and cyclin A.

Cyclin A abundance is periodically changed and tightly controlled during cell cycle. Posttranslational modifications such as phosphorylation, acetylation and ubiquitination play a key role in regulating cyclin A stability [27,28]. Previous studies indicate that E3 ubiquitin ligase complex APC/C^CDC20^ or APC/C^CDH1^ can promote ubiquitination of cyclin A for degradation [15,29]. Here, we found that DUB3 interacted with cyclin A and reversed cyclin A ubiquitination, thereby stabilizing cyclin A. Intriguingly, another member of the DUB family, USP37, also performs similar function on cyclin A and mediates similar regulation at the G1/S transition of the cell cycle. Since cyclin A is such an important factor in the determination of cell cycle progression, such multiple regulator mechanisms may contribute to ensuring its fine level during cell cycle.

It has been reported that DUB3 is overexpressed in a variety of human cancers including NSCLC. High levels of DUB3 is closely related to NSCLC recurrence and metastasis. Our results show that DUB3 mediates proliferation of NSCLC cells through stabilizing cyclin A. Knockdown of DUB3 inhibited cell proliferation. Furthermore, we found a strong correlation DUB3 and cyclin A in NSCLC samples. These findings suggest that DUB3 could be a potential target for NSCLC diagnosis and treatment.

## Supplementary Materials

The following are available online at https://www.mdpi.com/2073-4409/8/4/297/s1.
