# Supplementary material for: Deubiquitinase DUB3 Regulates Cell Cycle Progression via Stabilizing Cyclin A for Proliferation of Non-Small Cell Lung Cancer Cells
Source: Cells. 2019 Mar 31;8(4):297. doi: 10.3390/cells8040297 (PMC6523561; doi:10.3390/cells8040297)
Supplement: Supplementary file 1 [file cells-08-00297-s001.pdf]

Supplementary Figures

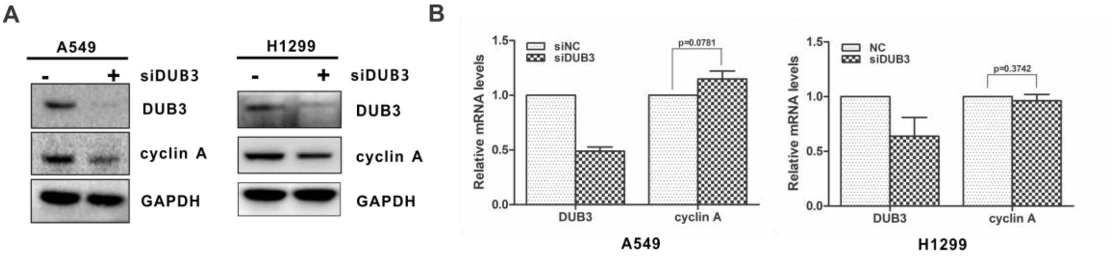

Figure S1. (A) A549 and H1299 cells were transfected with either scrambled or DUB3 siRNAs for 48 h. The resulting cell extracts were analyzed by Western blotting with anti-DUB3, anti-cyclin A, or anti-GAPDH antibody. (B) A549 and H1299 cells were transfected with either scrambled or DUB3 siRNAs for 48 h, and then total RNA was isolated and subjected to qRT-PCR. The error bars represent the SD of triplicate measurements.

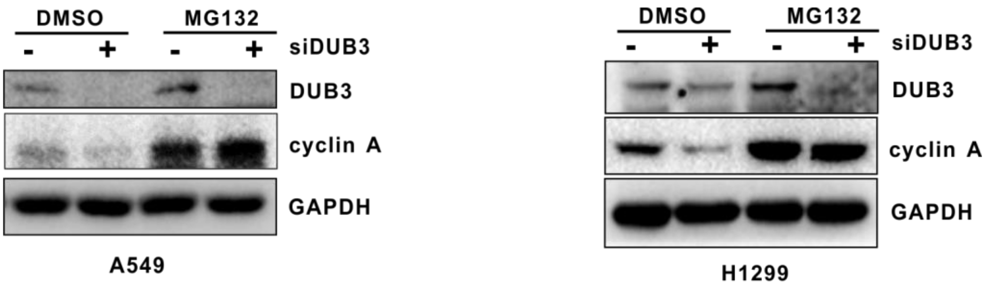

Figure S2 A549 and H1299 cells transfected with either scrambled or DUB3 siRNAs were treated with DMSO or MG132 (20μM) for 6 h, and the indicated proteins were analyzed byWestern blotting.

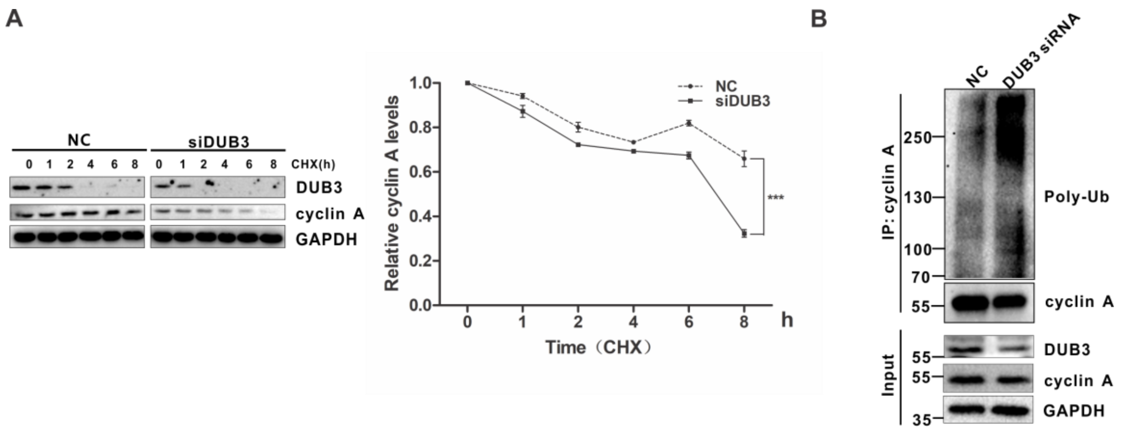

Figure S3 (A) A549 cells transfected with either scrambled or DUB3 siRNAs were treated with 50 μg·mL−1 CHX and then collected at the indicated time points for Western blot analysis. Quantification of the cyclin A levels relative to GAPDH expression is shown. Data represent the mean (± S.D.) of three independent experiments (*** $p < 0.001$). (B) A549 cells transfected with either scrambled or DUB3 siRNAs were treated with MG132 (20 μM) for 6 h before harvest. cyclin A was immunoprecipitated with an anti-cyclin A antibody, and the immunoprecipitates were probed with anti-Ub or anti-cyclin A antibody.

A

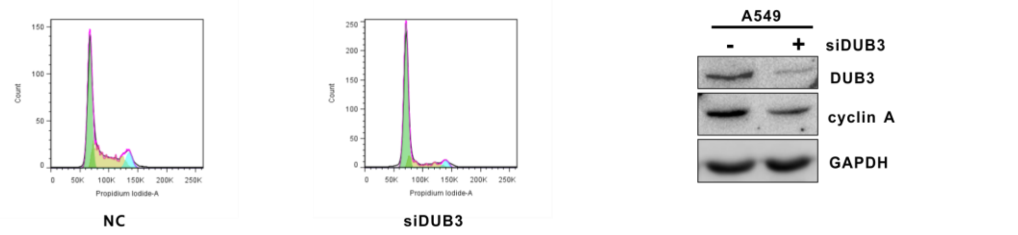

	NC	siDUB3
G0/G1 %	53.42±1.17	75.62±0.94
S%	34.38±1.13	18.25±0.29
G2/M%	12.21±0.04	6.13±1.23

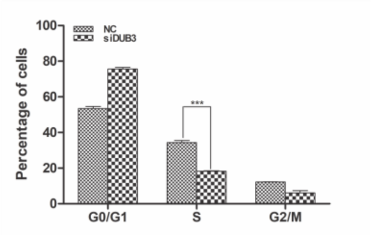

B

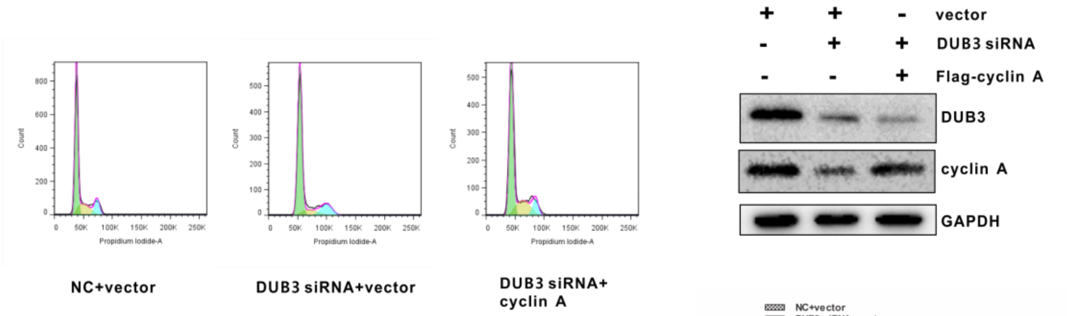

	NC+vector	siDUB3+vector	siDUB3+cyclin A
G0/G1 %	63.96±0.65	73.94±1.12	64.63±0.94
S%	24.14±1.54	11.2±1.68	24.18±2.08
G2/M%	11.9±0.89	14.86±0.56	11.19±1.14

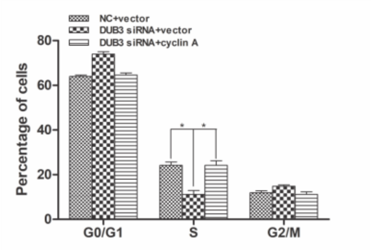

Figure S4. A549 cells transfected with either scrambled or DUB3 siRNAs were stained with propidium iodide and analyzed using flow cytometry. Data represent the mean (± S.D.) of three independent experiments (* $p < 0.05$ and *** $p < 0.001$).

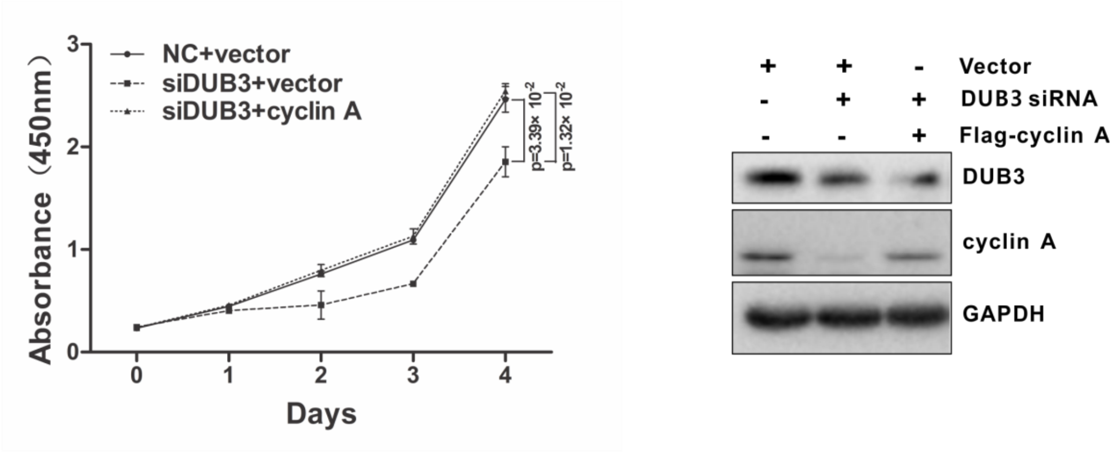

Figure S5. A549 cells were transfected with either scrambled or DUB3 siRNAs and then transfected with the indicated constructs. Cell proliferation was monitored using CCK-8 assays at the indicated time points. Statistical significance was determined by a two-tailed, unpaired Student’s t test.
